# Devascularized Bone Surface Culture: A Novel Strategy for Identifying Osteomyelitis-Related Pathogens

**DOI:** 10.3390/jpm12122050

**Published:** 2022-12-12

**Authors:** Peng Chen, Qing-rong Lin, Mou-Zhang Huang, Xin Zhang, Yan-jun Hu, Jing Chen, Nan Jiang, Bin Yu

**Affiliations:** 1 Division of Orthopaedics & Traumatology, Department of Orthopaedics, Southern Medical University Nanfang Hospital, Guangzhou 510515, China; 2 Guangdong Provincial Key Laboratory of Bone and Cartilage Regenerative Medicine, Southern Medical University Nanfang Hospital, Guangzhou 510515, China; 3 Department of Orthopaedics, Hainan General Hospital, Hainan Hospital affiliated to Hainan Medical University, Haikou 570311, China; 4 Department of Orthopaedics & Traumatology, Ganzhou Hospital Affiliated to Nanfang Hospital, Southern Medical University, Ganzhou 341099, China; 5 Department of Orthopaedics, Wuyi Hospital of Traditional Chinese Medicine, Jiangmen 523000, China; 6 Department of Laboratory Medicine, Southern Medical University Nanfang Hospital, Guangzhou 510515, China

**Keywords:** osteomyelitis, bone infection, bone surface culture, tissue sampling culture, *S. aureus*

## Abstract

The gold standard for identifying pathogens causing osteomyelitis (OM) is intraoperative tissue sampling culture (TSC). However, its positive rate remains inadequate. Here, we evaluated the efficiency of a novel strategy, known as devitalized bone surface culture (BSC), for detecting OM-related microorganisms and compared it to TSC. Between December 2021 and July 2022, patients diagnosed with OM and received both methods for bacterial identification were screened for analysis. In total, 51 cases were finally recruited for analysis. The mean age was 43.6 years, with the tibia as the top infection site. The positive rate of BSC was relatively higher than that of TSC (74.5% vs. 58.8%, *p* = 0.093), though no statistical difference was achieved. Both BSC and TSC detected definite pathogens in 29 patients, and their results were in accordance with each other. The most frequent microorganism identified by the BSC method was *Staphylococcus aureus*. Moreover, BSC took a significantly shorter median culture time than TSC (1.0 days vs. 3.0 days, *p* < 0.001). In summary, BSC may be superior to TSC for identifying OM-associated pathogens, with a higher detectable rate and a shorter culture time.

## 1. Introduction

Osteomyelitis (OM), also known as bone infection, is an inflammatory process following the invasion of pathogens, leading to inflammatory changes in osseous tissues [1]. It can occur following a contiguous focus, hematogenous spread, and vascular insufficiency [2]. Despite the great advances in the treatment, the clinical efficacy of OM remains inadequate, with an infection relapse rate ranging from 20% to 30% [3,4,5]. Such a high incidence of infection recurrence is associated with multiple factors [6], such as pathogen virulence, injury type, and treatment strategy.

The treatment of OM is complex and primarily depends on the initial causes and local pathological changes in patients [7]. Currently, treatment options include but are not limited to medullary space curettage, medullary reaming, medullary decompression, superficial decortication, sequestrectomy, soft tissue coverage, bone stabilization, and bone defect reconstruction [7]. Bone defects can be repaired by bone graft, Ilizarov technique, and Masquelet technique [1], or they may even be solved by utilizing bone tissue engineering strategies [8]. In addition to the aforementioned surgical interventions, one of the critical actions influencing the clinical efficacy of OM concerns antibiotic strategy, which is primarily on arthrocentesis and intraoperative sample cultures. Currently, standard intraoperative tissue sampling culture (TSC) is the gold standard for detecting the microorganisms which account for OM [9]. However, the positive rate of TSC remains inadequate, with most of the reported outcomes reaching around 60% [10]. Multiple factors affect its detectable rates, such as recent antibiotics and surgeries, the existence of bacteria biofilms, pathogen culture conditions, and sample selections [11]. Recently, different methods have been introduced and analyzed [10,12,13], aiming to increase the positive rate and guide the use of antibiotics.

In 2019, Moley and colleagues [14] reported using a novel method, the “agar candle dip”, to map the biofilms on the orthopedic explants. Inspired by this approach, we introduced a new strategy for the bacterial detection of fracture implant-associated infections (IAIs), known as “implant surface culture” (ISC) [15], based on the hypothesis that implant surfaces may be attached to bacterial biofilms. The outcomes of 42 patients demonstrated that the positive rate of ISC was significantly higher than that of TSC (85.7% vs. 54.8%, *p* = 0.002), signaling the definite efficiency of such a method as an adjunct treatment for bacterial identification purposes. Nonetheless, as mentioned in this study [15], one limitation is that it cannot be performed in the case of the retention of the implant hardware. In addition, there are still many OM patients without implants; thus, avenues to improve the detectable rate in such a group of patients require further exploration.

It is established that one of the typical histological characteristics of OM is the existence or formation of devascularized bone with or without sequestrum, which may provide a function for the attachment of bacteria biofilms [16], similar to those attached to the implants. Thus, we hypothesized that the direct culture on such object surfaces, referred to as “bone surface culture” (BSC), may increase the positive rate. Here, we compared the efficiency of BSC with TSC to detect OM-related microorganisms.

## 2. Materials and Methods

### 2.1. Study Setting, OM Diagnostic Criteria, and Inclusion and Exclusion Criteria

This prospective study was performed at Southern Medical University Nanfang Hospital, a tertiary medical center in Guangzhou, South China. The diagnosis of OM was referred to the diagnostic criteria of fracture-related infection (FRI) [17,18], including a sinus tract or fistula, wound breakdown or pus directly connecting the bone, visible pathogens measured via the histological test, and over five neutrophils per high power field (NP/HPF) [19]. The patients included were those with a confirmed diagnosis of OM following a contiguous focus or hematogenous spread, those with signed informed consent, those who stopped antibiotic use for at least two weeks, and those who received both methods for pathogen identification purposes. Patients were excluded if they were diagnosed with vascular insufficiency-related OM, joint infections, and prosthetic joint infections (PJIs) or received conservative treatment. In addition, patient data with any violations against prespecified BSC or TSC protocols were excluded. This study was conducted in line with the tenets of the 1964 Helsinki declaration and was approved by the Medical Ethical Committee of the Southern Medical University Nanfang Hospital (NFEC-2020-075).

### 2.2. BSC and TSC Procedures

All the included patients had stopped taking antibiotics for at least two weeks before surgery commenced. Intraoperative intravenous cephalosporins or clindamycin were administered only after the specimens were collected for culture and histology purposes. The same experienced surgeon collected the samples for both BSC and TSC.

**Figure 1:**
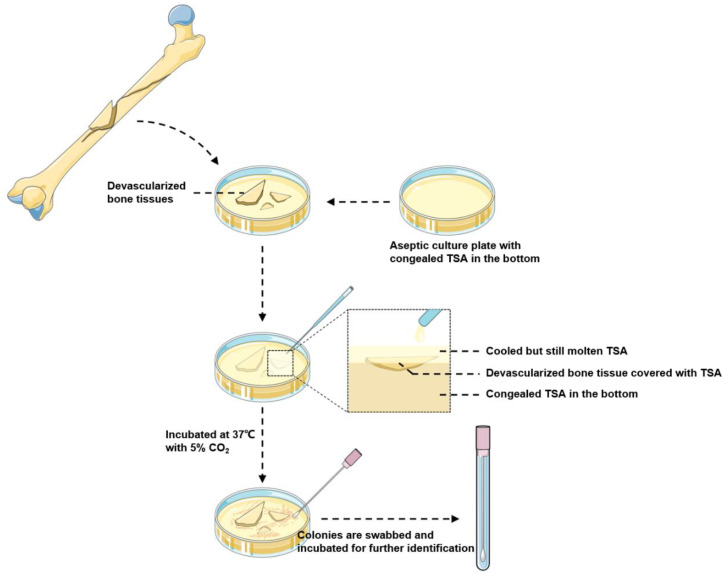
The schematic diagram of the BSC procedure.

The BSC procedure was similar to that of ISC [15]. First, the devascularized bone fragments, collected as much as possible, were directly set in an aseptic culture plate with congealed tryptic soy agar (TSA) at the bottom of the operation room. Then, the culture plate was transported to the biosafety cabinet of the laboratory within two hours. Then, the surface of the osseous tissue was gently covered with cooled and molten TSA. After that, the plate was incubated at 37 °C with 5% CO_2_. Sterile TSA was carefully added when necessary, to prevent the surfaces from drying out. The surfaces of the bone tissues and their surrounding culture media were examined every day for two weeks, as recommended [20], or until the colonies of the microorganisms appeared. If colonies were found, three different sites of colonies were separately swabbed and inoculated into the blood culture bottles. Then, the colonies were sampled by inoculating loops, and a mass spectrometer (Biomerieux, VITEK MS, Marcy-l’Étoile, France) was used for bacterial identification. A schematic diagram of the BSC procedure is depicted in Figure 1.

For TSC, the specimens from five different sites that were highly suspected of OM were collected. Then, the samples were disposed of by the working staff of the Clinical Laboratory within two hours. First, normal saline (10 mL) was used to homogenize the specimens separately with glass beads. Then, they were inoculated into the blood culture bottles (the BACTEC Lytic/10 Anaerobic/F bottle and the BACTEC Plus Aerobic/F bottle, Becton, Dickinson and Company, MD, Franklin Lakes, NJ, USA). The bottles were incubated at 37 °C with 5% CO_2_ for at least one week. Similarly, any identified colonies were collected by inoculating loops using the mass spectrometer (Biomerieux, VITEK MS, Marcy-l’Étoile, France) for bacterial identification purposes.

### 2.3. Statistical Analysis

The Statistical Package for Social Sciences software (version 17.0, SPSS Inc., Chicago, IL, USA) was used to conduct statistical analysis. The chi-square test was used to compare the positive rate, and a Wilcoxon signed-rank test was applied to compare the culture time, between the two methods. Statistical significance was defined as a *p*-value of ≤0.05.

## 3. Results

### 3.1. Participant Inclusion Flow Chart, Patient Demographics, OM Etiology, Body Side, and Infection Site Distributions

**Figure 2:**
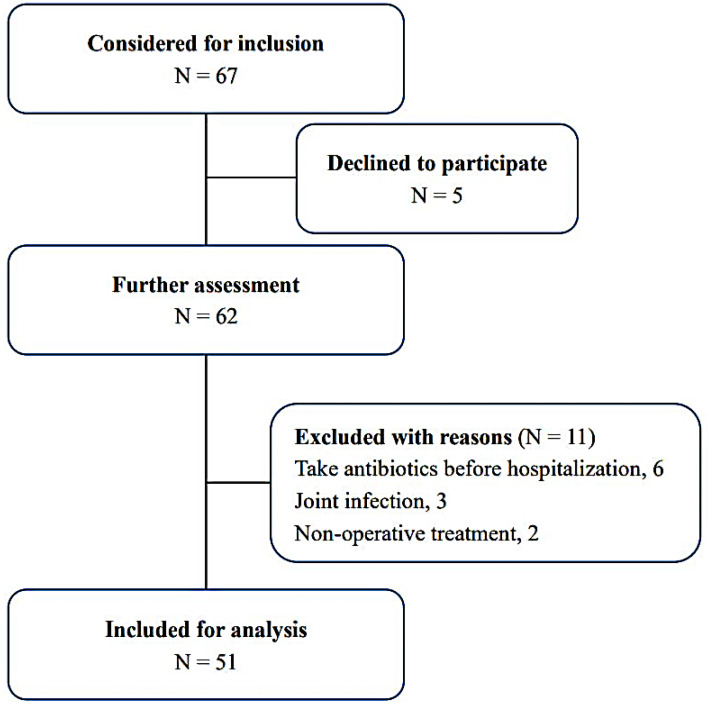
Eligibility selection process of the OM patients in this study.

A total of 67 patients were initially screened. After applying the inclusion and exclusion criteria, 51 patients (43 males) were finally included. A flow chart is depicted in Figure 2. The mean age of the included patients at diagnosis was 43.6 ± 17.4 years, with mean ages of 42.8 ± 17.0 years and 48.4 ± 20.0 years for males and females, respectively (*p* = 0.408). Among the 51 OM patients, 42 were classified as post-traumatic OM, with nine classified as hematogenous spread-related infection. Infections on the right body side were found in 26 cases, with 25 on the left side. The top three infected sites were the tibia (25 cases), calcaneus (11 cases), and femur (8 cases), followed by the humerus (3 cases), toes (2 cases), radius (1 case), and ulna (1 case) (Table 1).

### 3.2. BSC and TSC Outcomes and Culture Time

**Figure 3:**
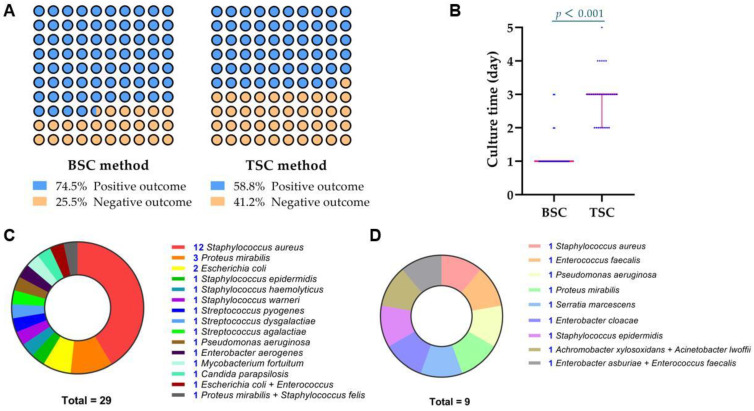
Graphical representation of the primary outcomes of the current study. Panel (**A**): Positive rates of the BSC and TSC strategies. Panel (**B**): The culture time of the BSC group was shorter than TSC group (a Wilcoxon signed-rank test, *p* < 0.001). Panel (**C**): Distribution of the microorganisms detected by both BSC and TSC, with consistent results. Panel (**D**): Distribution of the microorganisms identified by only BSC while TSC showed negative outcomes.

**Figure 4:**
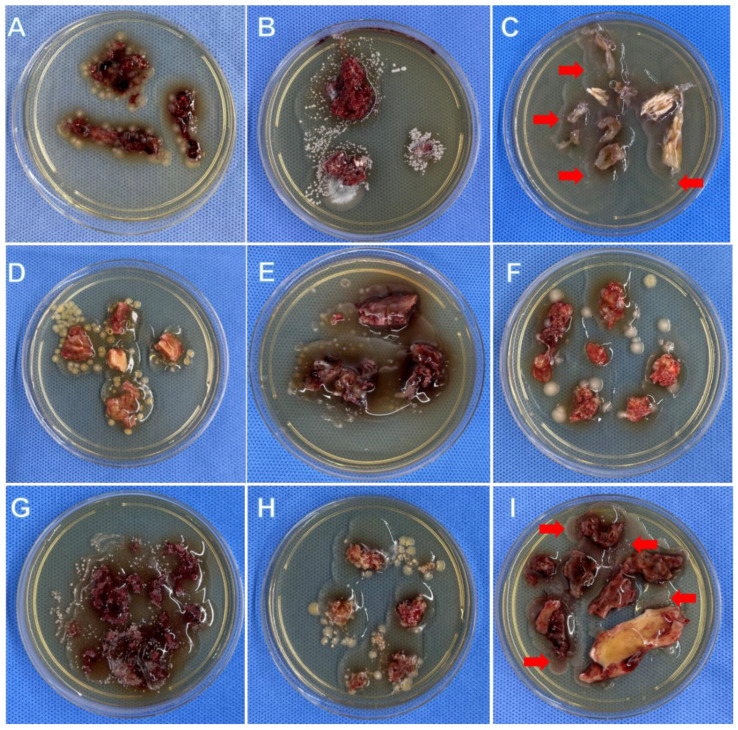
Nine OM patients showed positive outcomes using the BSC method. Panel (**A**): 49-year-old, male, tibia OM, BSC: *Escherichia coli*, TSC: *Escherichia coli.* Panel (**B**): 32-year-old, female, BSC: *Candida parapsilosis*, TSC: *Candida parapsilosis*. Panel (**C**): 71-year-old, male, tibia OM, BSC: *Proteus mirabilis*, TSC: *Proteus mirabilis* (see the arrows). Panel (**D**): 50-year-old, male, tibial OM, BSC: *Staphylococcus aureus*, TSC: *Staphylococcus aureus*. Panel (**E**): 16-year-old, male, femoral OM, BSC: *Enterococcus faecalis*, TSC: Negative. Panel (**F**): 42-year-old, male, tibia OM, BSC: *Enterobacter cloacae*, TSC: Negative. Panel (**G**): 39-year-old, male, tibia OM, BSC: *Mycobacterium fortuitum*, TSC: *Mycobacterium fortuitum*. Panel **(H**): 47-year-old, male, calcaneal OM, BSC: *Staphylococcus aureus*, TSC: *Staphylococcus aureus*. Panel (**I**): 43-year-old, male, femoral OM, BSC: *Enterobacter aerogenes*, TSC: *Enterobacter aerogenes* (see the arrows).

**Figure 5:**
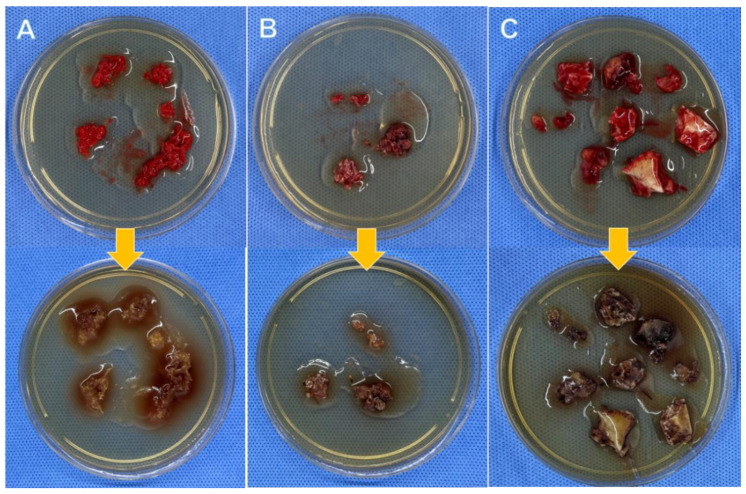
Three OM patients revealed negative outcomes by the BSC method (images in the upper row: the initial stage of BSC; images in the lower row: the end stage of BSC). Panel (**A**): 15-year-old, male, tibia OM, TSC: negative. Panel (**B**): 32-year-old, male, tibia OM, TSC: negative. Panel (**C**): 37-year-old, male, tibia OM, TSC: negative.

The total positive rate of BSC (38/51) was relatively higher than TSC (30/51), though no statistical difference was found (74.5% vs. 58.8%, *p* = 0.093). Both BSC and TSC detected definite pathogens in 29 patients, and their results were in accordance with each other. In addition, BSC took a significantly shorter median culture time than TSC (1.0 days vs. 3.0 days, *p* < 0.001). The graphical representation of the primary outcomes of the present study is depicted in Figure 3. The detailed results regarding both of the methods are presented in Table 1. Figure 4 shows nine patients with positive outcomes, whereas Figure 5 displays three patients with negative outcomes, measured via the BSC method.

### 3.3. Microorganism Type

The BSC method detected 38 patients with definite pathogens, and 34 were identified as having monomicrobial infections. The most frequent pathogen was *Staphylococcus aureus* (13 cases), followed by *Proteus mirabilis* (4 cases), *Pseudomonas aeruginosa* (2 cases), *Escherichia coli* (2 cases), and *Staphylococcus epidermidis* (2 cases). Another 11 types of microorganisms were only found in a single patient (Table 1), including a fungus, *Candida parapsilosis*.

## 4. Discussion

As mentioned previously, aside from surgery, the timely, effective, and correct identification of OM-related pathogens is one of the most critical measures that can be taken to decrease the risk of infection recurrence and improve treatment efficacy. However, currently, the efficiency of TSC remains inadequate. According to a recent multicenter study in Northeast China [21], the positive rate of traditional culture among FRI patients was only 50.8%, which is far from satisfying. To increase the detection rate, several novel strategies of culture have been reported and analyzed, such as sonication fluid culture [12] and culture from the reamer–irrigator–aspirator (RIA) system [13]. In a prospective cohort study, Finelli et al. [12] compared the efficiency between traditional peri-implant tissue culture and sonication fluid culture in patients with intramedullary nailing infection. The outcomes of 54 patients revealed that the positive rates of conventional culture and sonication fluid culture were similar (89.4% vs. 97.6%), while the sonication fluid culture displayed advantages in identifying polymicrobial infections. In addition to the sonication fluid culture, Onsea et al. [13] introduced a strategy of cultures from the RIA system. The results from 24 patients indicate that such a novel method displayed similar efficiency when compared to the standard tissue culture (71% vs. 67%). The current BSC method also revealed similar diagnostic accuracy with the RIA system culture (74.5% vs. 71%) but was inferior to the sonication fluid culture. Nonetheless, all three novel strategies had advantages over TSC.

Recently, Moley et al. [14] reported using an “agar candle dip” method to map biofilms on orthopedic explants. In light of this study, we tried to cover the culture medium on the surfaces of the explants among patients with IAIs and achieved satisfactory outcomes [15]. However, one intrinsic limitation of this method is that it cannot be conducted among patients without implants, which limits its application. Here, we expanded and modified this method and directly poured TSA on the devascularized bone surface, which resembled an implant surface, where many great biofilms might also become attached. Outcomes demonstrated that the delay of positivity was hugely reduced with the BSC method compared to TSC, suggesting that BSC is also a valuable approach for detecting OM-related pathogens, especially those without implants. We believe that one primary reason accounting for the superiority of BSC over TSC is that all the available devascularized bone tissues are collected and cultured, lowering the risk of selection bias to the minimum.

Although the current BSC strategy is referred to as the “agar candle dip” method, the efficiency of BSC appears to be superior. It can be primarily attributed to several possible factors. First, Moley and colleagues used different types of materials from the prosthesis for culture purposes, while we only cultured the devascularized bone tissue. Secondly, they used brain heart infusion (BHI) agar, whereas we used TSA agar. The case of whether the culture medium influences the detection efficiency requires further investigation. Thirdly, Moley et al. incubated the components for seven days, while we extended such a culture time to 14 days. Of course, the decision regarding whether 14-day incubation is necessary needs to be evaluated in the future. Lastly, they included only 15 patients for analysis, which may also affect the outcomes.

The present BSC technique shares similarities and differences with ISC. For similarities, firstly, both BSC and ISC used the same culture medium, TSA, to cover the in vitro tissues and implants. As one of the most frequently used culture media, TSA acts as a general-purpose non-selective medium providing abundant nutrients which allow a wide variety of microorganisms to grow and can also be used for the storage, maintenance, and transportation of pure pathogens. However, the case of whether TSA is the optimal medium requires further analysis. Secondly, the culture conditions and duration of the two methods are the same, both with incubation at 37 °C under 5% CO_2_, with a consecutive run of 14 days or until the appearance of colonies of the microorganisms. Third, as both of the methods require TSA agar supplementation during culture, repeated exposures of the tissues or implants increase the contamination risk. Therefore, on the one hand, necessary controls should be set to identify whether contaminations occur or not clearly. On the other hand, procedures and tools may be modified to lower such risks to the minimum.

Regarding differences, firstly, we did not rinse the devascularized bone tissues with normal saline before culture, as we would like to keep the original situation or status of the deep tissues and lower the contamination risk. The case of whether rinsing is an essential procedure for BSC needs further exploration. Secondly, our study was different from ISC as there was nothing on the bottom of the culture plate. Meanwhile, for BSC, already congealed TSA was prepared in advance in the bottom before the osseous tissues were placed. The primary reason is that, quite different from explants; the bone tissues may lose vitality and quickly become dry without the medium in the bottom. Thirdly, BSC may display a higher risk of selection bias than ISC. For ISC, the whole implants are obtained and covered with TSA. Whereas the selection of devascularized bone tissues largely relies on the surgeons’ experiences with BSC, though the specimens have been selected and cultured as much as possible, selection bias cannot be avoided entirely. Further within-person comparisons should be conducted to evaluate the efficacy levels between BSC and ISC.

Interestingly, *Candida parapsilosis* was found by BSC in a 32-year-old female patient with OM following trauma, and the TSC result also confirmed such a type of fungi. Fungal OM and septic arthritis are rare, with *Candida* and *Aspergillus* being the most frequent agents [22]. In 2016, Gamaletsou et al. [23] summarized the clinical features, diagnosis, and treatment of *Candida*-related arthritis based on the synthesis of reported cases within the literature. The outcomes of 112 patients demonstrated that *Candida*-related arthritis primarily affects the hips and the knees. Despite antifungal therapy, the successful treatment of such an infection still poses significant challenges. In addition to the case of fungi-related infection, *Mycobacterium fortuitum* was detected by both methods in a 39-year-old male diagnosed with tibia OM. It is known that *Mycobacterium fortuitum* is a type of slow-growing bacterium which is often associated with contamination. OM related to *Mycobacterium fortuitum* is rare and was occasionally presented as a single case report. In 2015, Grantham et al. [24] reported OM related to *Mycobacterium fortuitum* in a 14-year-old patient following reconstruction surgery for the anterior cruciate ligament. The infection was successfully eradicated by repeated debridement and irrigation with systematic and local antibiotics. Recently, Fraga et al. [25] also presented a case diagnosed of OM associated with *Mycobacterium fortuitum* in the cuboid bone of a 61-year-old female. She was successfully treated by debridement in combination with the local implantation of calcium sulfate containing gentamicin and vancomycin.

It is also notable that one patient showed a negative result following BSC, while TSC showed infection associated with *S. aureus*. Such an unexpected result may be correlated with selection bias during sample collections. To further increase the detection rate, integrated and standardized sampling procedures for BSC should be established. Aside from sample selections, another factor that may lead to negative results is that both BSC and ISC cannot identify the anaerobic bacteria associated with the intrinsic limitation of the two strategies. In this study, we excluded patients with OM related to vascular insufficiency (e.g., diabetic foot OM), mainly because most of these patients usually have ulcers which may increase the risk of contamination.

In addition to the above-mentioned intrinsic limitations of the BSC method, our study also has several limitations. Firstly, the method reported in this paper is preliminary and requires further optimization. Moreover, the sample dispensing and culture strategy indeed have significant room for refinement and improvement. To better evaluate the efficiency of this novel strategy, a well-designed study with a larger sample size as well as necessary controls is warranted to calculate the sensitivity, specificity, and diagnostic accuracy of OM. Secondly, this study did not analyze the risk factors linked to the negative outcomes of BSC, which need to be further investigated. Thirdly, although both BSC and ISC have shown satisfying results, the better of the two remains to be seen as their direct comparisons are lacking. Fourthly, we did not assess therapeutic efficacy as the follow-up time was short. Thus, future studies should focus on the treatment efficacy and its potential influencing factors.

## 5. Conclusions

In conclusion, our study suggests that BSC may be an effective and valuable strategy for identifying microorganisms that cause OM, with a higher positive rate and a shorter culture time. Procedures of this method should be optimized to increase the detection rate of OM-related pathogens.

## Figures and Tables

**Table 1:** Clinical features of the included OM patients, culture outcomes, and culture time by BSC and TSC.

Case No.	Sex/Age (Year)	Infection Site	BSC Outcome	BSC Time (Day)	TSC Outcome	TSC Time (Day)
1	M/14	Femur	*Staphylococcus aureus*	1	*Staphylococcus aureus*	3
2	M/29	Tibia	*Staphylococcus aureus*	1	*Staphylococcus aureus*	3
3	M/41	Tibia	*Achromobacter xylosoxidans + Acinetobacter lwoffii*	3	Negative	NA
4	M/49	Tibia	*Escherichia coli*	1	*Escherichia coli*	3
5	F/49	Calcaneus	Negative	NA	Negative	NA
6	M/44	Tibia	*Staphylococcus epidermidis*	3	Negative	NA
7	M/53	Femur	*Staphylococcus aureus*	1	*Staphylococcus aureus*	3
8	M/14	Tibia	*Streptococcus pyogenes*	1	*Streptococcus pyogenes*	2
9	F/32	Tibia	*Candida parapsilosis*	1	*Candida parapsilosis*	2
10	M/71	Tibia	*Proteus mirabilis*	1	*Proteus mirabilis*	2
11	M/59	Femur	*Proteus mirabilis*	1	*Proteus mirabilis*	2
12	M/10	Phalange	*Staphylococcus epidermidis*	1	*Staphylococcus epidermidis*	3
13	M/53	Calcaneus	*Proteus mirabilis + Staphylococcus felis*	1	*Proteus mirabilis + Staphylococcus felis*	5
14	M/51	Calcaneus	*Staphylococcus aureus*	1	*Staphylococcus aureus*	4
15	M/59	Radius	*Staphylococcus aureus*	1	Negative	NA
16	M/21	Humerus	Negative	NA	Negative	NA
17	M/50	Tibia	*Staphylococcus aureus*	1	*Staphylococcus aureus*	2
18	M/49	Tibia	*Staphylococcus aureus*	1	*Staphylococcus aureus*	4
19	M/16	Femur	*Enterococcus faecalis*	1	Negative	NA
20	F/68	Tibia	Negative	NA	Negative	NA
21	M/68	Calcaneus	*Proteus mirabilis*	1	*Proteus mirabilis*	3
22	F/54	Calcaneus	Negative	NA	Negative	NA
23	M/46	Calcaneus	*Staphylococcus aureus*	1	*Staphylococcus aureus*	3
24	F/48	Femur	*Staphylococcus aureus*	1	*Staphylococcus aureus*	4
25	M/42	Tibia	*Enterobacter cloacae*	1	Negative	NA
26	M/37	Tibia	Negative	NA	Negative	NA
27	M/39	Tibia	*Mycobacterium fortuitum*	3	*Mycobacterium fortuitum*	3
28	M/87	Humerus	*Pseudomonas aeruginosa*	3	*Pseudomonas aeruginosa*	3
29	M/52	Calcaneus	Negative	NA	Negative	NA
30	M/47	Calcaneus	*Staphylococcus aureus*	1	*Staphylococcus aureus*	3
31	M/15	Tibia	Negative	NA	Negative	NA
32	M/29	Phalange	*Staphylococcus aureus*	1	*Staphylococcus aureus*	2
33	M/36	Femur	Negative	NA	*Staphylococcus aureus*	3
34	M/52	Tibia	*Pseudomonas aeruginosa*	3	Negative	NA
35	M/56	Tibia	*Staphylococcus warneri*	2	*Staphylococcus warneri*	3
36	M/32	Tibia	Negative	NA	Negative	NA
37	M/67	Calcaneus	*Escherichia coli*	1	*Escherichia coli*	4
38	M/29	Tibia	*Staphylococcus haemolyticus*	1	*Staphylococcus haemolyticus*	3
39	M/45	Calcaneus	Negative	NA	Negative	NA
40	M/37	Tibia	Negative	NA	Negative	NA
41	M/49	Ulna	*Serratia marcescens*	1	Negative	NA
42	M/66	Tibia	*Staphylococcus aureus*	1	*Staphylococcus aureus*	2
43	M/25	Tibia	*Staphylococcus aureus*	1	*Staphylococcus aureus*	3
44	M/48	Calcaneus	*Streptococcus agalactiae*	1	*Streptococcus agalactiae*	4
45	F/63	Tibia	*Streptococcus dysgalactiae*	2	*Streptococcus dysgalactiae*	3
46	M/30	Tibia	*Proteus mirabilis*	1	Negative	NA
47	M/43	Femur	*Enterobacter aerogenes*	1	*Enterobacter aerogenes*	3
48	M/49	Tibia	Negative	NA	Negative	NA
49	F/8	Tibia	Negative	NA	Negative	NA
50	M/30	Femur	*Escherichia coli + Enterococcus*	1	*Escherichia coli + Enterococcus*	2
51	F/65	Humerus	*Enterobacter asburiae + Enterococcus faecalis*	2	Negative	NA

OM: osteomyelitis; BSC: Bone surface culture; TSC: tissue sampling culture; NA: not available.

## Data Availability

Not applicable.
